# Meigs’ syndrome mimicking heart failure with preserved ejection fraction: a case report

**DOI:** 10.1186/s12872-020-01718-4

**Published:** 2020-10-07

**Authors:** Yoko Murayama, Yoshiro Kamoi, Hiroyuki Yamamoto, Jun Isogai, Takahiro Tanaka

**Affiliations:** 1 Department of Cardiology, Cardiovascular Center, Showa General Hospital, Tokyo, Japan; 2 Department of Cardiovascular Medicine, Narita-Tomisato Tokushukai Hospital, 1-1-1 Hiyoshidai, Tomisato, Chiba, 286-0201 Japan; 3 Department of Radiology, Asahi General Hospital, Asahi, Japan

**Keywords:** Heart failure with preserved ejection fraction, Meigs’ syndrome, Mitotically active cellular fibroma of the ovary, Pleural effusion, Exudative effusion

## Abstract

**Background:**

Meigs’ syndrome is a rare disease characterized by a triad of presentations, including benign ovarian tumor, ascites, and pleural effusion. However, a clinical diagnosis of Meigs’ syndrome remains challenging because pleural and ascitic effusions can be common findings in a variety of underlying conditions. Furthermore, these findings can often be misdiagnosed as pleural and peritoneal dissemination caused by potentially malignant tumors, leading to the administration of improper treatment.

**Case presentation:**

We described a case of an 85-year-old postmenopausal female patient with atypical Meigs’ syndrome presenting with right-sided pleural effusion, notable leg edema, and trivial ascites, which was initially mistaken as heart failure with preserved ejection fraction. However, pleural effusion was totally ineffective against diuretic therapy. Subsequently, thoracentesis yielded serosanguineous exudative effusion. Moreover, refractory pleural effusions and abdominal/pelvic computed tomography and magnetic resonance imaging findings strongly suggested bilateral malignant ovarian tumors with pleural dissemination. Repetitive negative cytological results allowed the patient to undergo bilateral salpingo-oophorectomy. Finally, a definitive diagnosis of Meigs’ syndrome was made by confirming the presence of a benign mitotically active cellular fibroma of the ovary by pathology and that pleural effusion resolved following tumor resection.

**Conclusions:**

Our case highlights the clinical importance of assessing Meigs’ syndrome in the diagnostic workup of pleural effusion in postmenopausal female patients. Given the favorable prognosis of Meigs’ syndrome, clinicians should consider surgical resection, even with potentially malignant ovarian tumors with accompanying pleural effusion, ascites, or both.

## Background

Meigs’ syndrome (MS) is an uncommon disorder characterized by the clinical triad of presentations including, benign ovarian tumor, ascites, and pleural effusion (PE), which disappear after tumor resection [1]. MS occurs in approximately 1% of all ovarian tumors. It is most commonly associated with ovarian fibromas, identified in 2–5% of surgically resected ovarian tumors [2]. MS is most frequently diagnosed in postmenopausal women, especially those around 50 years of age [3]. Presenting symptoms include fatigue, dyspnea, dry cough, weight loss, and abdominal distension. Due to non-specific symptoms and signs, many patients with MS are initially referred to general practitioners. Thus, a correct diagnosis is often postponed, and appropriate patient treatment is delayed in cases of potential malignant ovarian tumors in terms of systemic tumor cell dissemination.

## Case presentation

An 85-year-old menopausal woman was referred to our hospital for diagnostic evaluation of worsening dyspnea over 6 weeks. The patient had a history of chronic atrial fibrillation, hypertension, and chronic kidney disease (CKD) of uncertain etiology. She had a uterine fibroid diagnosed at 64 years of age. Pre-admission medication included amlodipine (10 mg/day), valsartan (40 mg/day), verapamil (120 mg/day), digoxin (0.125 mg/day), and rivaroxaban (10 mg/day).

**Fig. 1:**
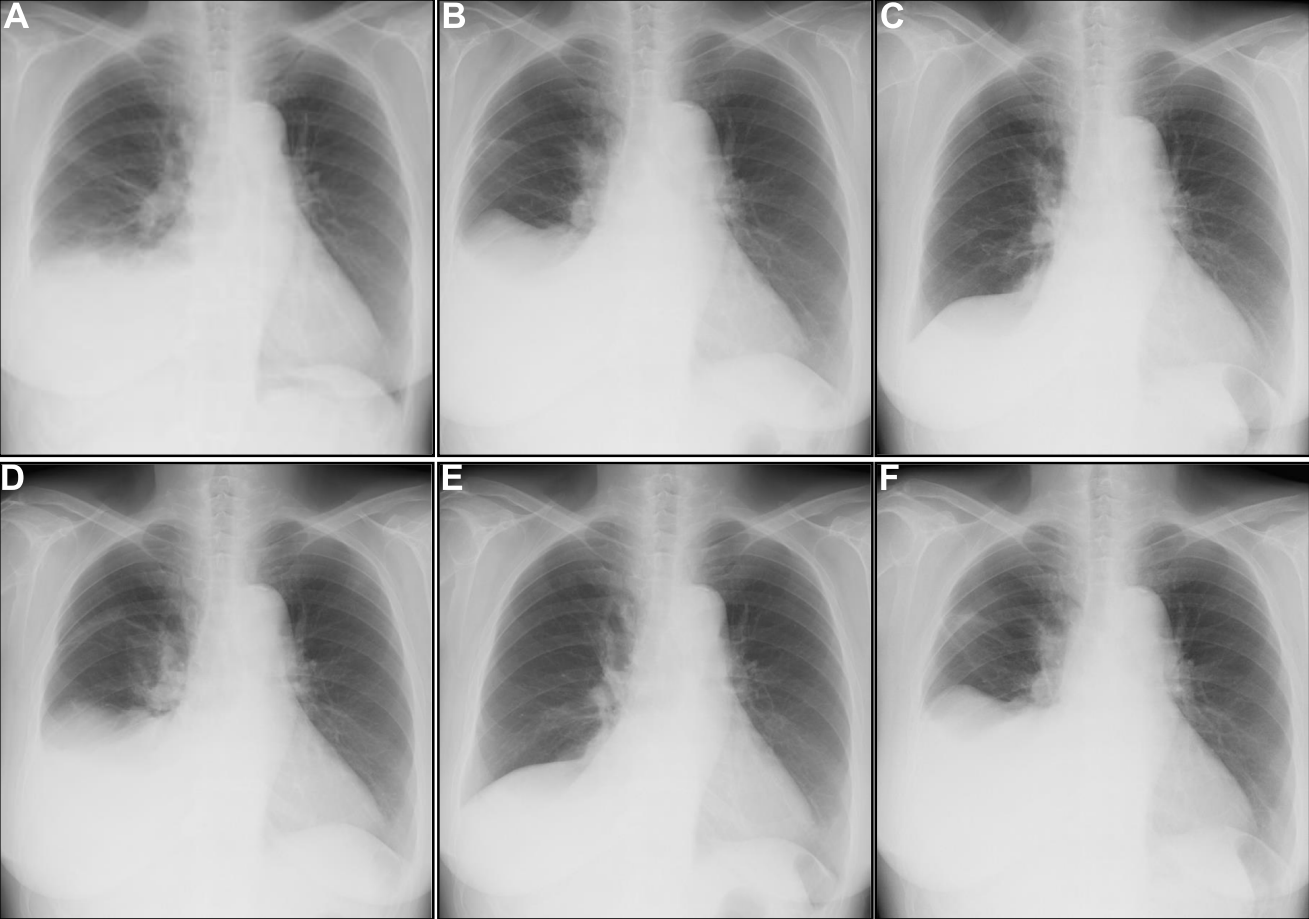
Serial chest radiographs. **a** On admission; an initial radiograph shows right-sided pleural effusion. **b** Day 6; radiograph following diuretic therapy. Note, the effusion remains unchanged. **c** Day 7; radiograph after the removal of 1240 mL effusion via the first thoracentesis shows resolution of effusion. **d** Day 14; follow-up radiograph after first thoracentesis shows reaccumulation of effusion. **e** Day 16; radiograph after the removal of 1200 mL effusion via a second thoracentesis shows resolution of effusion. **f** Day 23; follow-up radiograph following second thoracentesis shows reaccumulation of effusion

Her vital signs were as follows: blood pressure 146/97 mmHg, heart rate 90 beats/min and irregular, and respiratory rate 21 breaths/min. Physical examination revealed a third heart sound, diminished breath sounds from the right lower lungs, and notable bilateral leg edema, but no remarkable jugular vein distention (JVD) was observed. A hard mass was palpable in the lower abdomen. The electrocardiogram revealed atrial fibrillation. Chest radiography revealed cardiomegaly and right-sided PE (Fig. 1a). Laboratory testing revealed elevated levels of serum creatinine (1.36 mg/dL, reference, 0.65–1.07 mg/dL) and brain natriuretic peptide (BNP) (183 pg/mL, reference: < 18.4 pg/mL). Hepatic and thyroid function, C-reactive protein levels, and urinalysis were within normal ranges.

Echocardiography demonstrated normal left ventricular (LV) cavity size and systolic function, with an ejection fraction of 54% and left atrial dilation (35 mL/m^2^) (Additional files 1 and 2). Doppler and tissue Doppler profiles for the assessment of LV diastolic function revealed a decreased septal e′ of 5.8 cm/sec and an elevated E/e′ of 17, indicating elevated LV filling pressure. In addition, continuous wave Doppler revealed a peak tricuspid regurgitation velocity of 2.6 m/s, consistent with a pressure gradient of 27 mmHg and an estimated right ventricular systolic pressure of approximately 35 mmHg. Echocardiographic evaluation of the right heart revealed almost normal systolic function; tissue Doppler of the free lateral wall (S′) = 9 cm/s (reference: ≥ 10 cm/s), tricuspid annular plane systolic excursion = 18 mm (reference: 16 ≥ mm), and fractional area change = 53% (reference: ≥ 35%), respectively.

Thus, a tentative diagnosis of heart failure with preserved ejection fraction (HFpEF) was made. The patient was administered oxygen at 3 L/min and treated with intravenous loop diuretics (furosemide 40 mg daily). On day 6, although diuretic treatment improved leg edema, PE remained unchanged (Fig. 1b).

A subsequent diagnostic workup of unexplained PE was performed. The patient underwent thoracentesis with drainage of 1240 mL of serosanguineous PE. PE analysis revealed lymphocyte-predominant exudates fulfilling Light’s criteria (Table 1). Additionally, the serum-to-effusion albumin gradient was 0.3 g/dL (reference: ≤ 1.2 g/dL), further confirming the true exudative effusion. The adenosine deaminase activity in PE was 9.3 U/L. PE cytology, as well as the bacterial and mycobacterial cultures, were unremarkable.

**Table 1:** Patient’s laboratory data

Variables	Results	Reference range
**Blood analysis**		
Total protein (g/L)	6.7	6.3–8.2
Albumin (g/L)	3.2	3.4–5.0
LDH (U/L)	134	106–211
Tumor markers		
CA-125 (U/mL)	382	0–35
CEA (ng/mL)	2.1	< 5.0
NSE (ng/mL)	8.2	< 16.3
**Pleural fluid**		
Color	reddish	
Rivalta test	negative	
White blood cells (/μL)	627	
Neutrophils (%)	1	
Lymphocytes (%)	69	
Histiocytes (%)	28	
Eosinophils (%)	0	
Protein (g/L)	4.9	
Albumin (g/L)	2.9	
LDH (U/L)	104	
Cholesterol (mg/dL)	44	
Glucose (mg/dL)	134	
Adenosine deaminase (U/L)	9.3	
Culture: M. tuberculosis	negative	
Cytology	class II	
Identification of exudative effusions		
Protein fluid to serum ratio	0.73	> 0.5
LDH fluid to serum ratio	0.77	> 0.6
Serum-to-effusion albumin gradient (g/dL)	0.3	≤ 1.2

*LDH* Lactate dehydrogenase, *CA-125* Cancer antigen-125, *CEA* Carcino-embryonic antigen, *NSE* Neuron-specific enolase, *M. tuberculosis Mycobacterium tuberculosis*

**Fig. 2:**
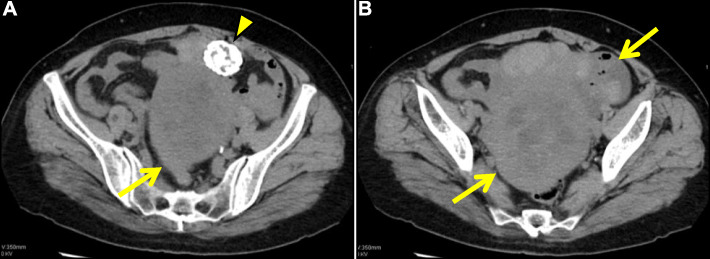
Non-contrast-enhanced abdominal/pelvic computed tomography. **a** and **b**: Axial images show bilateral inhomogeneous and well-circumscribed ovarian masses (arrows). Note the huge soft tissue mass with a central low attenuation area in the pouch of Douglas, which is bordered ventrally by a calcified uterine fibroid (arrowhead)

**Fig. 3:**
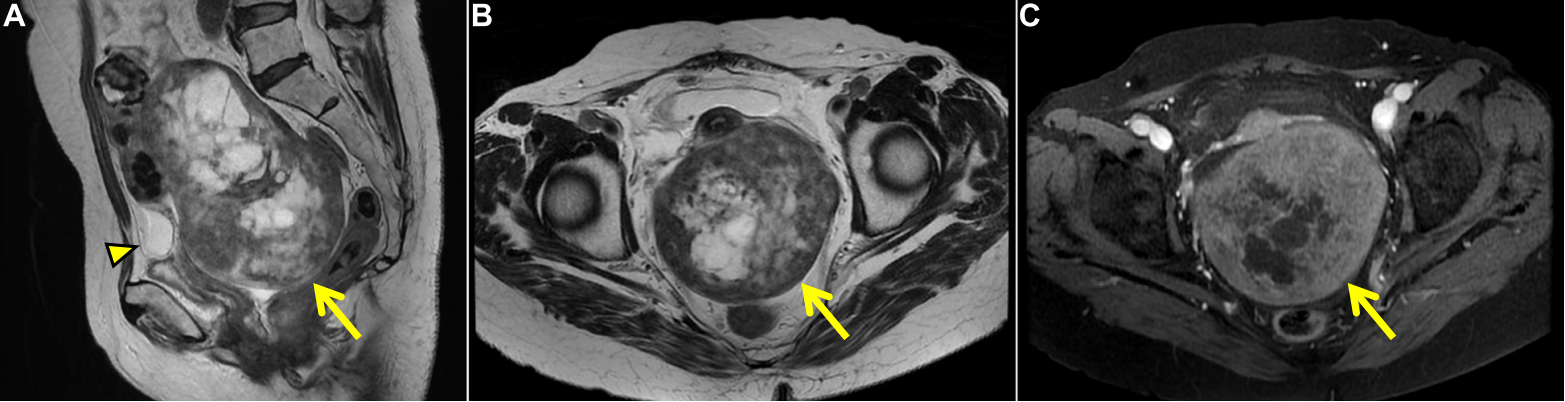
Pelvic magnetic resonance imaging (MRI). **a** Sagittal and **b** axial T2-weighted, and **c** axial gadolinium-enhanced fat-suppressed T1-weighted images. MRI shows the right huge ovarian mass in 14-cm of the long axis, with a multilocular cystic component in the central tumor, and demonstrates the peripheral solid portion as an obvious hypointense area on T2-weighted image and slight and inhomogeneous gadolinium enhancement (arrows). A trivial ascites in the vesico-uterine pouch is also observed (arrowhead)

Chest computed tomography revealed an absence of lung tumors or inflammatory infiltration after the removal of PE. Abdominal/pelvic computed tomography scan revealed bilateral inhomogeneous ovarian masses (Fig. 2). Magnetic resonance imaging further characterized the right substantial mass and detected a trivial ascites (Fig. 3). Axial and sagittal T2-weighted images demonstrated the peripheral hypointense mass, which contained hyperintense and multilocular areas in the central portion, mimicking a malignant ovarian tumor with a central necrosis or a degenerated subserosal fibroid. Gadolinium-enhanced axial imaging showed inhomogeneous enhancement correspondently on the peripheral solid portion, suggesting malignancy. In addition, screening tests for tumor markers revealed elevated CA-125 levels (382 U/mL, reference: < 35 U/mL). Repeated serial thoracentesis was ineffective, and PE reaccumulated within a week (Fig. 1c-f). Thus, we highly suspected of ovarian cancer with pleural dissemination. Nevertheless, a repeated cytological examination of PE revealed no evidence of malignancy, which led us to consider the possibility of MS.

**Fig. 4:**
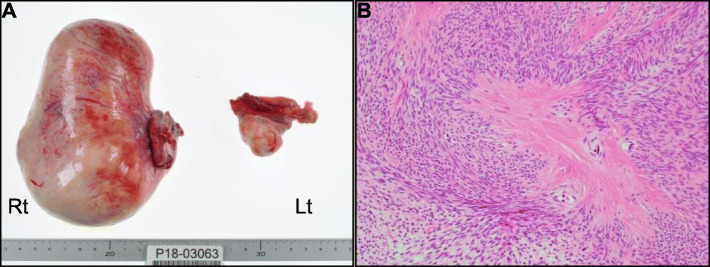
Macroscopic image of resected ovarian tumors and pathological findings. **a** Resected masses. The right resected ovarian mass measures 13 × 8 × 8 cm in size and weighed 643 g. Rt, right; Lt, left. **b** Photomicrograph after hematoxylin and eosin staining (× 100). Dense fibroblast-like cellular proliferation, formed in intersecting bundles is observed

On day 26, the patient underwent bilateral salpingo-oophorectomy (Fig. 4). The resected masses exhibited yellow-to-tan fleshy cut surfaces, with hemorrhage and extensive hyaline degeneration. Histopathological examination revealed a mitotically active cellular fibroma (MACF) of the ovary. Spontaneous resolution of the right-sided PE was noted post-operatively after 7 days of follow-up, confirming a definitive diagnosis of MS. A follow-up echocardiography revealed no significant changes. The post-operative course was uneventful, and the patient made a full recovery and was discharged with no changes to her pre-admission medication regimen on day 33. She remained clinically stable upon subsequent follow-up.

## Discussion and conclusions

Typically, MS presents with right-sided PE and ascites in the presence of a benign ovarian tumor (e.g., fibroma). MS is common in middle-aged to elderly postmenopausal women. On the contrary, there are some reported cases of atypical MS with either PE or ascites and bilateral PE without ascites [4].

PE is a common finding caused by a wide variety of diseases and conditions. The leading causes of PE include chronic heart failure (HF), chronic renal failure, malignancy, pneumonia, and pulmonary embolism; thus, PE caused by such conditions requires differentiation from MS-associated PE. Our case also exhibited atypical MS manifestations, characterized by right-sided PE, notable leg edema, and trivial ascites, which was initially mistaken as HFpEF.

HFpEF is characterized by signs and symptoms of HF with preserved LV ejection fraction (≥ 50%) and evidence of diastolic dysfunction. Epidemiologically, the ratio of patients with HFpEF increases strikingly with age, especially in postmenopausal women having multiple noncardiac comorbidities, such as obesity, hypertension, CKD, and diabetes [5]. HFpEF is gaining considerable attention because of rapid population aging around the world [6].

Although MS and HFpEF are similar in terms of gender and age of onset and are accompanied PE, they differ in terms of prerequisite treatment strategies. Thus, MS-associated PEs must be clinically differentiated from HF-associated PEs. In our case here, PEs were non-responsive to diuretics, and bilateral leg edema was notable despite the absence of JVD; the findings suggested venous stasis yield by ovarian tumors. These mismatched findings might be a crucial key to the diagnosis of the uncommon disease presentation observed in our patient. Presumably, despite the evidence for LV diastolic dysfunction, mildly elevated circulating BNP levels are considered to be attributable to physiological elevation caused by aging and CKD.

Second, the serum-to-effusion albumin gradient was more useful in identifying exudative effusion in patients receiving diuretic therapy than were Light’s criteria [7]. A systematic review of the literature demonstrated that the majority of MS-associated PEs are exudates [3]. The mode of MS-associated PE is significantly right-sided (70%), followed by being bilateral (19%) and left-sided (11%). On the contrary, HF-associated PEs are transudates, and the mode of HF-associated PE is predominantly bilateral (64%), followed by being right-sided (24%) and left-sided (12%) [8]. Detailed analyses, including those for biochemical characteristics of PE, can provide valuable information concerning properties, etiologies, and possible mechanisms involved in PE formation. PE analysis is the first diagnostic approach for PE of unknown etiology, and Light’s criteria can distinguish transudate effusion and exudative effusion [7]. However, there is a pitfall in the evaluation of Light’s criteria because of enrichment of the protein or lactate dehydrogenase component of the PE caused by diuretic use. In fact, 29% of HF-associated PEs are misclassified as exudative in HF patients receiving diuretic therapy [9]. The serum-to-effusion albumin gradient has superior diagnostic value in detecting true exudative effusions compared with Light’s criteria (sensitivity, 87%; specificity, 92% and sensitivity, 98%; specificity, 83%, respectively) [7]. Moreover, the serum-to-effusion albumin gradient allowed for a correct diagnosis in our patient.

Although the etiology of PE and ascites remains unknown, MS has been considered to be a subset of porous diaphragm syndrome, characterized by a congenital or acquired anatomical defect, typically in the right hemidiaphragm, through which ascites can passage into the right-sided pleural space [10]. Another possible mechanism is that proinflammatory cytokines cause increased vascular permeability and capillary leakage. In fact, considerably elevated levels of vascular endothelial growth factor and interleukin-6 have been observed in serum, ascitic effusions, and PEs of patients with MS [11]. Moreover, the evidence that the majority of MS-associated PEs are exudative, strongly support this hypothesis [3]. However, we could not measure levels of any proinflammatory cytokines in the present case.

Finally, repeated cytological examination was helpful in the diagnosis of MS.

MACF of the ovary is a rare histopathologic entity; it is defined as a benign ovarian cellular fibrous tumor with high mitotic activity without severe atypia, has a different behavior from the distinct malignant behavior of ovarian fibrosarcoma, and is associated with a favorable outcome [12]. Owing to the high count of mitotic figures, MACF often provides diagnostic challenges in cases of pelvic mass with ascites, PE, and elevated tumor marker CA-125 levels. Nevertheless, our patient, after repeated benign cytologic findings, did undergo successful surgical treatment. Thus, this case highlights the clinical importance of MACF in the diagnostic workup of a potential malignant ovarian tumor. While its prognosis is considered generally favorable, poor prognostic factors, including postoperative residual tumor tissue, peritoneal adhesion, and capsular rupture, have been reported [13]. Long-term monitoring is needed for patients with MACF, since the natural history remains poorly understood.

Overall, herein, we described a case of MACF with MS mimicking HFpEF.

MS is extremely rare, yet frequently causes PE and ascites. The correct diagnosis and appropriate treatment are often missed, since a variety of diseases are also associated with the development of these common signs. Therefore, all clinicians should be aware of the clinical significance of MS in the diagnostic assessment of PE in postmenopausal female patients, and PE analysis and abdominal screening are warranted. Given the favorable prognosis of MS, clinicians should consider the possibility of performing surgical resection, even if a potentially malignant pelvic mass with PE, ascites, or both, are present.

## Supplementary information

**Additional file 1.** Transthoracic echocardiography in an apical 4-chamber view.

**Additional file 2.** Transthoracic echocardiography in a parasternal short-axis view.

## Data Availability

Data sharing is not applicable to this article as no datasets were generated or analyzed during the current study.

## Abbreviations

MS: Meigs’ syndrome
PE: Pleural effusion
CKD: Chronic kidney disease
JVD: Jugular vein distention
BNP: Brain natriuretic peptide
LV: Left ventricular
HFpEF: Heart failure with preserved ejection fraction
MACF: Mitotically active cellular fibroma
HF: Heart failure

## References

[1] Meigs JV (1954). Fibroma of the ovary with ascites and hydrothorax; Meigs’ Syndrome. Am J Obstet Gynecol.

[2] Saha S, Robertson M (2012). Meigs’ and Pseudo-Meigs’ syndrome. Australas J Ultrasound Med.

[3] Krenke R, Maskey-Warzechowska M, Korczynski P, Zielinska-Krawczyk M, Klimiuk J, Chazan R, Light RW (2015). Pleural Effusion in Meigs’ Syndrome-Transudate or Exudate?: systematic review of the literature. Medicine (Baltimore).

[4] Taniguchi Y, Nishikawa H, Maeda N, Terada Y (2020). Breathlessness, Pleural Effusions, Fibromas, and Meigs Syndrome: Look Beyond the Chest and Don’t Delay!. Lancet.

[5] Mentz RJ, Kelly JP, von Lueder TG, Voors AA, Lam CS, Cowie MR, Kjeldsen K, Jankowska EA, Atar D, Butler J (2014). Noncardiac comorbidities in heart failure with reduced versus preserved ejection fraction. J Am Coll Cardiol.

[6] Andersen MJ, Borlaug BA (2014). Heart failure with preserved ejection fraction: current understandings and challenges. Curr Cardiol Rep.

[7] Richard W (2002). Light. Clinical practice. Pleural effusion. N Engl J Med.

[8] Camilla L (2009). Wong, Jayna Holroyd-Leduc, Sharon E Straus. Does this patient have a pleural effusion?. JAMA..

[9] Roth BJ, O’Meara TF, Cragun WH (1990). The serum effusion albumin gradient in the evaluation of pleural effusions. Chest..

[10] Kirschner PA (1998). Porous diaphragm syndromes. Chest Surg Clin N Am.

[11] Abramov Y, Anteby SO, Fasouliotis SJ, Barak V (2001). Markedly elevated levels of vascular endothelial growth factor, fibroblast growth factor, and interleukin 6 in Meigs syndrome. Am J Obstet Gynecol.

[12] Matsuda K, Tateishi S, Akazawa Y, Kinoshita A, Yoshida S, Morisaki S, Fukushima A, Matsuwaki T, Yoshiura K-I, Nakashima M (2016). Rapid growth of mitotically active cellular fibroma of the ovary: a case report and review of the literature. Diagn Pathol.

[13] Bucella D, Limbosch JF, Buxant F, Simon P, Fayt I, Anaf V, Noël JC (2009). Recurrence of mitotically active cellular fibroma of the ovary. Obstet Gynecol Int.

